# Identification and Analysis of Long Non-Coding RNAs Related to UV-B-Induced Anthocyanin Biosynthesis During Blood-Fleshed Peach (*Prunus persica*) Ripening

**DOI:** 10.3389/fgene.2022.932207

**Published:** 2022-08-09

**Authors:** Man Zhang, Xiuqi Zhang, Haijing Wang, Mao Ye, Yating Liu, Zhihua Song, Tingting Du, Hongyan Cao, Liqin Song, Xiao Xiao, Jianzhen Liu, Libin Zhang, Yangbo Song, Qing Yang, Dong Meng, Junkai Wu

**Affiliations:** ^1^ College of Horticulture Science and Technology, Hebei Normal University of Science and Technology, Qinhuangdao, China; ^2^ Hebei Key Laboratory of Horticultural Germplasm Excavation and Innovative Utilization, Qinhuangdao, China; ^3^ The Key Laboratory for Silviculture and Conservation of Ministry of Education, The College of Forestry, Beijing Forestry University, Beijing, China; ^4^ College of Agriculture and Animal Husbandry, Qinghai University, Xining, China

**Keywords:** blood-fleshed peach, lncRNAs, UV-B, anthocyanin biosynthesis, ripening

## Abstract

Blood flesh is a key fruit trait in peaches (*Prunus persica*) and can be attributed to the accumulation of anthocyanins. The roles of long non-coding RNAs (lncRNAs) have been highlighted by multiple studies in regulating fruit ripening, anthocyanin accumulation, and abiotic stress responses in many flowering plants. Such regulatory functions of lncRNAs in Prunus persica, nonetheless, have not been reported. In this research, we sequenced and analyzed the complete transcriptome of C3-20 (a blood-fleshed peach) fruit at four developmental stages. Analyses of the correlated genes and differentially expressed lncRNA target genes helped to forecast lncRNAs’ possible functions. The RNA-seq data were generated using high-throughput sequencing. In total, 17,456 putative lncRNAs, including 4,800 intergenic lncRNAs, 2,199 antisense lncRNAs, and 10,439 intronic lncRNAs were discovered, of which 4,871 differentially expressed lncRNAs (DE-lncRNAs) were annotated in the fruit developmental processes. The target genes of these DE-lncRNAs and their regulatory relationship identifying 21,795 cis-regulated and 18,271 trans-regulated targets of the DE-lncRNAs were in a similar way predicted by us. The enriched GO terms for the target genes included anthocyanin biosynthesis. Flavonoid biosynthesis and plant hormone signal transduction were also included in the enriched KEGG pathways. Co-expression network construction demonstrated that the highly expressed genes might co-regulate multiple other genes associated with auxin signal transduction and take effect in equal pathways. We discovered that lncRNAs, including LNC_000987, LNC_000693, LNC_001323, LNC_003610, LNC_001263, and LNC_003380, correlated with fruit that ripened and could take part in ethylene biosynthesis and metabolism and the ABA signaling pathway. Several essential transcription factors, such as *ERFs, WRKY70, NAC56*, and *NAC72*, may in a similar way regulate fruit ripening. Three DE-lncRNAs, XLOC_011933, XLOC_001865, and XLOC_042291, are involved in UV-B-induced anthocyanin biosynthesis and positively regulating *UVR8* and *COP10,* were identified and characterized. Our discovery and characterization of XLOC_011933, XLOC_001865, and XLOC_042291 provide a more precise understanding and preliminarily establishes a theoretical framework for UV-B-induced flesh anthocyanin biosynthesis. This phenomenon might encourage more in-depth investigations to study the molecular mechanisms underlying peach flesh coloring.

## Introduction

Peach, a small deciduous tree, belongs to the order Rosales, family Rosaceae, subfamily Prunoidae, genus *Prunus*, and *Prunus persica* (L.) Batsch. According to the color of their flesh, there are three main types of peaches in the market, namely, white peach, yellow peach, and red peach. The book *Luoyanghuamuji* contained records of red peaches with blood color. The flesh peach first appeared in Europe in 1,659 (Hedrick, 1917). Accumulation of anthocyanins leads to attractive blood-fleshing in peaches and nectarines (He et al., 2015).

Anthocyanins, which generate characteristic reddish, bluish, and purple hues, are essential determinants of the color of many plant organs (Welch et al., 2008; Escribano-Bailón et al., 2004; Mano et al., 2007; Espley et al., 2007; Deluc et al., 2008). The anthocyanin content is an important indicator of ripening because many fruits and vegetables do not accumulate anthocyanins until they are ripening (Jimenez-Garcia et al., 2013). Anthocyanins also have potential human health benefits and represent a necessary aspect of fruit quality (Martin et al., 2011). The genetics and biochemistry of the anthocyanin accumulation-mediated fruit coloration and its biosynthetic pathway have been well studied. Different anthocyanins include various anthocyanidin aglycones. Among these, six anthocyanidins, Cy, Dp, Pg, Pn, Pt, and Mv, are generally found in most fruits (Macheix et al., 1990). In peach fruits, anthocyanins’ predominant component is cyanidin-3-glucoside, with amounts of cyanidin-3-rutinoside. Many botanists have made significant contributions to the study of anthocyanin biosynthesis to develop new varieties with high anthocyanin content (Xie et al., 2011).

Anthocyanin biosynthetic pathways are well known to be conserved in many species (Grotewold, 2006; Lin-Wang et al., 2010; Lim and Ha, 2013). This well-known pathway acts via the phenylpropanoid pathway leading to the formation of anthocyanins, in which a series of enzymes are located on the endoplasmic reticulum’s cytoplasmic surface, including *CHS*, *CHI*, *F3H*,*F3′H*,*F3′5′H*, *DFR*, *LDOX,* and *UFGT* (Holton and Cornish, 1995). Several studies have explained the pathway of anthocyanin biosynthesis in detail. However, it is essential to identify other critical players in such complicated regulatory networks.

A variety of environmental factors participate in and influence the biosynthesis process of anthocyanin, resulting in a series of changes in the anthocyanin content (Guo et al., 2008). As we all know, light is a major influencing factor in anthocyanin biosynthesis in the plant kingdom (Albert et al., 2009; Lin-Wang et al., 2011; Azuma et al., 2012; Butelli et al., 2012). Light can positively elevate the fruit anthocyanin content based on its characteristics, including specific light quality and light intensity photoperiod (Ubi et al., 2006; Li et al., 2013). Fruits produce anthocyanins to effectively scavenge the reactive oxygen species (ROS) produced in response to excess UV light. Anthocyanin enhancement in apple skin has been observed after UVB irradiation (Peng et al., 2013; Zoratti et al., 2014). Specific classes of plant photoreceptors, including *PHYs*, *CRYs*, *PHOTs,* and *UVR8*, allow plants to sense the presence of light and thus regulate the biosynthesis of secondary metabolites (Rizzini et al., 2011). Downstream signaling elements of photoreceptors, such as *COP1* and *HY5,* can also be further activated (Stracke et al., 2010; Lau and Deng, 2012). The gene of *COP1* is a key negative regulator of light signal transduction and participates in plant growth under light irradiation. *COP10* is, in a similar way, crucial for photomorphogenesis. *Arabidopsis AtCOP10* was found to be an inhibitor of the transcription factor HY5’s COP1-mediated degradation within the nucleus (Osterlund et al., 2000). *HY5* is inconsistent with the two factors mentioned previously and is considered to be a positive regulatory gene with light involvement (Lee et al., 2007). *HY5* is the main gene controlled by *COP1* in dark environments (Osterlund et al., 2000). The expression of *HYH*, which changes the same with the expression of the anthocyanin biosynthesis pathway’s regulation of structural genes, is correlated with the anthocyanin content (Zhao et al., 2017). A total of 12 light receptors (*UVR8s*, eight LIGHT-DEPENDENT SHORT HYPOCOTYLS) and four constitutive photomorphogenesis proteins (*COP*) were derived by automated computational analysis in the peach genome.

Long non-coding RNAs (lncRNAs) are characterized by a transcription length of more than 200 nt and not coding proteins (Ma et al., 2013). LncRNAs play essential regulatory roles in various biological processes, such as developmental and environmental regulation (Liu et al., 2012; Wen et al., 2007; Wang M. et al., 2015; Wang T.-Z. et al., 2015; Xin et al., 2011; Li et al., 2007; Boerner and McGinnis, 2012; Tang et al., 2016), *Hippophae rhamnoides* Linn (Zhang et al., 2017), and *Populus* (Liu et al., 2018). LncRNAs were expressed during pollen tube germination and pollen tube growth in plants (Kim and Sung, 2012). Increasing evidence suggests that lncRNAs play essential roles in regulating secondary metabolism (An et al., 2018; Yin et al., 2018). However, the profiles of lncRNAs in fruit trees are not clear. Many research studies have shown that lncRNAs participate in fruit ripening. Another study on tomatoes implicated the silencing of lncRNA1459 and lncRNA 1840, leading to a conspicuous delay in the wild-type fruit’s ripening (Wang et al., 2018). LncRNA1459 functional loss mutant produced by the Cas9 gene-editing technique showed that the tomato ripening process, ethylene production, and lycopene accumulation were primarily repressed, and the expression of the ripening-related genes was significantly altered, providing clues for understanding the function of lncRNA1459 in the ripening of tomato fruit (Li et al., 2014; Zhu et al., 2015). In apples, eight kinds of lncRNAs associated with fruits were tested and found to regulate fruit ripening and glucose metabolism (An N. 2018). RNA sequencing analysis identified DE-lncRNAs in the red and yellow sea buckthorns’ fruit, providing a resource for the in-depth study of fruit coloring in ripe fruits (Zhang et al., 2017). In kiwi fruits, lncRNAs associated with fruit development and ripening have been identified (Tang et al., 2016).

Although the role of lncRNAs in various biomolecular processes has been gradually discovered, the regulatory role of lncRNAs in Rosaceae trees is still poorly understood, particularly in peaches. Peaches (*Prunus persica*) are economically important fruit trees with a short maturation phase and a sequenced genome (2*n* = 16, 225.7 Mb) (Verde et al., 2017). The regulatory mechanism of anthocyanins in peaches with the blood-flesh phenotype has been extensively researched, combining high-quality sequenced genomes. The key genes that regulate the phenotype of the flesh are well known, including *MYB, bHLH, WD40,* and TFs that form the *MYB-bHLH* or *MYB-bHLH-WD40* (*MBW*) complex. However, whether there are other regulatory factors affecting anthocyanin biosynthesis remains to be researched. The present study analyzed blood-flesh transcriptomes of peach fruits at different developmental stages to screen for lncRNAs related to anthocyanin biosynthesis to further understand the regulatory network of the blood-flesh phenotype in peach flesh. In total, 17,456 lncRNAs were identified in the blood-fleshed peach fruit transcriptome dataset. Expression correlation was used between mRNAs and lncRNAs in the peach reference genome. Both positive and negative lncRNA–mRNA pairs were identified. XLOC_011933, XLOC_001865, and XLOC_042291, which are involved in UV-B-induced anthocyanin biosynthesis and positively regulate *UVR8* and *COP10* (constitutive photomorphogenic 10), were identified and characterized. Our investigation served as a reference for later studies exploring the function of lncRNAs in red peaches.

## Materials and Methods

### Sample Collection, RNA Quantification, and Qualification

The blood-fleshed hybrid lines of the *Prunus persica* (L.) Batsch “C3-20” seedling was grown in the experimental station of Hebei Normal University of Science and Technology, Hebei Province, China. Ovary samples were collected 10 days before and 10 days after pollination. The samples were collected from some 5-year-old peaches and designated as follows: 34 DAP, 44 DAP, 54 DAP, 64 DAP, and 74 DAP (DAP refers to days after pollination). The samples we collected, including fruit and ovary, are wrapped in tin foil and stored in a −80°C refrigerator, which is convenient for RNA extraction and physiological indicator measurement.

### RNA Isolation, Library Preparation, and Sequencing

Total RNA was isolated by applying the TRIzol reagent (Invitrogen, Carlsbad, CA, United States). The RNA quality was determined by agarose gel electrophoresis. The integrity of the total RNA was also determined. In addition, each sample was constructed with 3 μg RNA. First, ribosomal RNA was removed using a kit (epicenter, United States). Subsequently, the NEBNext®Ultra™ Directed RNA Library preparation kit from Illumina^®^ (NEB, United States) was used.

### RNA-Seq Read Processing, Mapping, and Transcriptome Assembly

The authenticity of the quality of sequencing results was checked. We used HISAT2 for the comparative analysis of the reference genome of the filtered reads (Langmead and Salzberg, 2012, 2). StringTie used the results of HISAT2 alignment to splice transcripts, resulting in the smallest possible set of transcripts (Pertea et al., 2016). After screening the lncRNA and TUCP transcripts, the StringTie-EB software was used for the quantitative analysis of the transcripts including mRNA, lncRNA, and TUCP (Trapnell et al., 2010; Pertea et al., 2016).

### Identification of lncRNAs

In the initial stage, Cuffmerge software was used to merge the transcripts obtained (Trapnell et al., 2010). Then, the combined transcript set was screened for lncRNA: in the first step, the exon number of transcripts greater than or equal to 2 was selected; in the second step, the length of the transcripts >200 bp was retained; the third step is the screening of known annotations in the transcript; the fourth step is to select transcripts with FPKM greater than or equal to 0.5; and the last step is the screening of coding potential (Kong et al., 2007; Cabili et al., 2011; Lin et al., 2011; Punta, et al., 2012; Sun et al., 2013).

### Target Gene Prediction

LncRNAs can interact with target genes in either *cis* or *trans* form, so we adopted two methods to predict the *target* genes of lncRNAs. The *cis* role shows the lncRNA’s effect on its neighboring target genes. The coding genes were screened in the 10 kb upstream and downstream regions of lncRNAs. The *trans* role indicates the ability of the lncRNAs to identify each other based on individual expression levels. As the number of samples was less than 25, the Pearson correlation coefficient was used to further analyze the correlation between lncRNAs and the expression level of protein-coding genes. When the absolute value of the correlation coefficient was greater than 0.95, we believed that there was trans-interaction between protein-coding genes and lncRNAs.

### GO and KEGG Enrichment Analysis

The GO enrichment analysis method was GOseq and it was performed using the GOseq R package (Young et al., 2010). KEGG is a major public database related to pathways. The pathway significance enrichment analysis was conducted with the KEGG pathway as the unit (Mao et al., 2005). The KEGG (http://www.genome.jp/kegg/) enrichment analysis was performed by KOBAS (2.0) software. The interactions between differential lncRNAs and mRNAs were analyzed using Cytoscape software to construct a correlation network diagram (Saito et al., 2012).

### Construction of a DE-lncRNA–mRNA Network

The networks of the lncRNA–mRNAs were to elucidate the functions of lncRNAs. Based on the interaction relations in the STRING protein interaction database (http://string-db.org/), the interaction relations of target gene sets (such as the differential gene list) were directly extracted from the database to construct the network for the species contained in the database.

### Determination of the Total Anthocyanin Content

We used methanol supplemented with 1% HCl from the plant material. The samples were ground in N2 solution and cultured overnight. Then, after centrifugation for 15 min at 16,000 × *g*, the absorption value of the material at 530 and 657 nm was determined by using a spectrophotometer. Finally, the following formula was used to calculate the anthocyanin content in each period: *Q*_anthocyanins_ = (*A*_530_−0.25 × *A*_657_) × M^−1^; Q_anthocyanins_ content of anthocyanins, *A*_530_ absorption at 530 nm wavelength, *A*_657_ absorption at 530 nm wavelength, and *M* fresh weight (g) of the tissues (Mehrtens et al., 2005).

### Quantitative Real-Time (qRT)-PCR

TransStart Tip Green qPCR SuperMix (Transgen) and CFX Connect were used for qRT-PCR (Supplementary Table S6). The reference gene for qRT-PCR was the peach *RPL13* (ribosomal protein L13) gene. The results were unified using the 2^−ΔΔt^ calculation method.

### Statistical Analysis

All experiments were set up as three replicates, and each data were represented by error lines. All data in this experiment were plotted and analyzed by GraphPad Prism 9, where *p* < 0.05 was * and *p* < 0.01 was ***. The online website (http://www.bioinformatics.com.cn) drawing heatmaps and enrichment analysis diagrams was used.

## Results

### Library Construction and RNA Sequencing of Different Development Stages of Blood-Fleshed Peach

**FIGURE 1:**
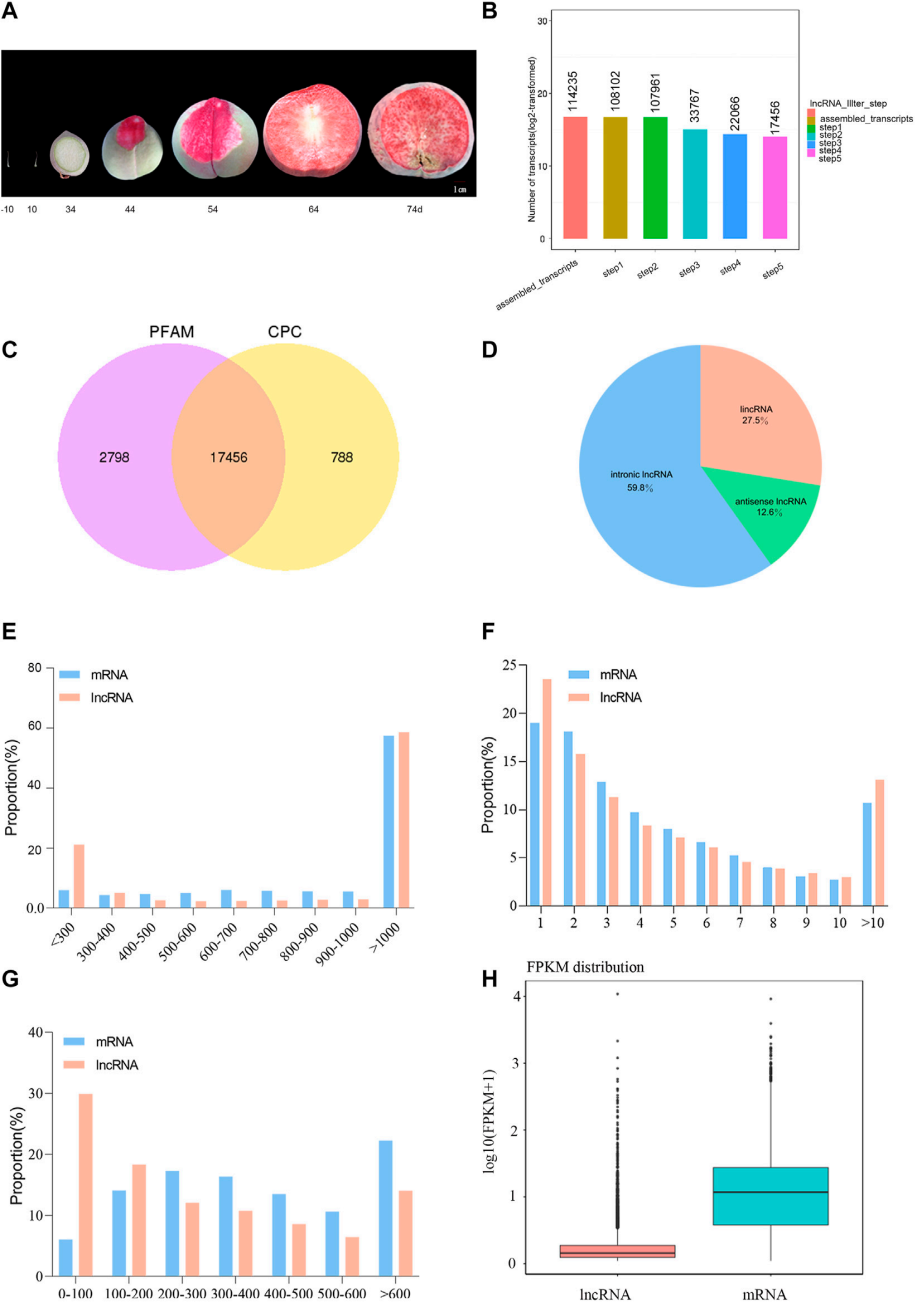
General information on the lncRNAs in peach. **(A)** Phenogram of the peach. **(B)** Basic screening charts for lncRNAs. **(C)** Screening results using CPC and Pfam. **(D)** Composition of different types of lncRNAs. **(E)** Length distribution of peach lncRNAs and mRNAs. **(F)** Distribution of some exons in lncRNAs and mRNAs. **(G)** Distribution of the ORFs in lncRNAs and mRNAs. **(H)** Comparison of expression levels between lncRNAs and mRNAs.

Ovaries were collected 10 days before pollination and 10 DAP, and peaches were harvested at 34, 44, 54, 64, and 74 DAP (Figure 1A). The percentage of clean reads ranged from 96.66–98.48% (Table 1). The Q20 and Q30 values were >97 and 92%, respectively, which proved that the quality control data are reliable. The GC content ranged between 43.99 and 44.69%. We could clearly observe that the mapping rate of clean reads was 80.62–90.69% and most of the clean reads (77.7–87.6%) were distributed in the protein-coding region (Supplementary Tables S1–S2). A total of 114,235 assembled transcripts were used to screen for lncRNAs. After five basic screening steps (described in Section 2.4), 17,456 transcripts were retained, which were used to analyze protein-coding potential using CPC and PFAM (PfamScan) (Figures 1B,C). According to their locations in the *P. persica* genome, 4,800 lincRNAs (27.5%), 2,199 antisense lncRNAs (12.6%), and 10,439 intronic lncRNAs (59.8%) were identified (Figure 1D). The composition of different lncRNAs is different from that of *Populus* lncRNAs (Liu et al., 2018). The structural analysis of lncRNAs and mRNAs indicated that those in peach fell in the length range of 201–20,369 and 3–15,738 nt, respectively, with corresponding averages of 1,623 and 1,321 nt, respectively. The average transcript length of the lncRNAs was slightly more than that of the mRNAs, although the difference was not as significant as that in poplar (Liu et al., 2018). Most lncRNAs and mRNAs were >1,000 bp long (58.59 and 57.41%, respectively). The distribution of exon numbers showed a similar trend for the lncRNAs and mRNAs (Figure 1F). For example, 23.51% of the lncRNAs had one, 15.79% had two, 11.31% had three, 19.02% had one, 18.08% had two, and 12.88% had three exons (Figure 1F). The open reading frames (ORFs) of lncRNAs were 22–5,114 nucleotides (nt) in length, of which the majority (29.89%) were ≤100 nt. The ORFs of the mRNAs were 1–5,245 in length, with the majority accounting for 22.64%, being ≥600 nt (Figure 1G). The expression level of most lncRNAs was relatively low (Figure 1H).

**TABLE 1:** Sequencing quality.

Sample name	Raw reads	Clean reads	Percent (%)	Error rate (%)	Clean bases (G)	Q20 (%)	Q30 (%)	GC content (%)
S1_11	103,290,726	100,842,110	97.63	0.02	15.13	97.14	92.75	44.11
S1_12	107,939,668	105,585,708	97.82	0.01	15.84	97.51	93.83	44.45
S1_13	98,090,490	96,262,152	98.14	0.01	14.44	98.03	94.94	44.1
S2_21	105,311,206	103,705,758	98.48	0.01	15.56	97.89	94.63	43.99
S2_22	115,876,152	113,957,100	98.34	0.01	17.09	97.9	94.64	44.16
S2_23	97,715,896	95,926,382	98.17	0.01	14.39	97.99	94.83	44.29
S3_31	104,871,196	102,758,664	97.99	0.01	15.41	97.91	94.66	44.38
S3_32	86,772,210	83,870,212	96.66	0.01	12.58	97.98	94.74	44.36
S3_33	99,187,418	96,244,550	97.03	0.01	14.44	97.96	94.68	44.44
S4_41	110,074,586	106,839,978	97.06	0.01	16.03	98.09	94.97	44.57
S4_42	106,348,746	103,571,376	97.39	0.01	15.54	98	94.77	44.69
S4_43	100,381,514	97,653,486	97.28	0.01	14.65	97.94	94.65	44.67

### Differentially Expressed lncRNAs

**FIGURE 2:**
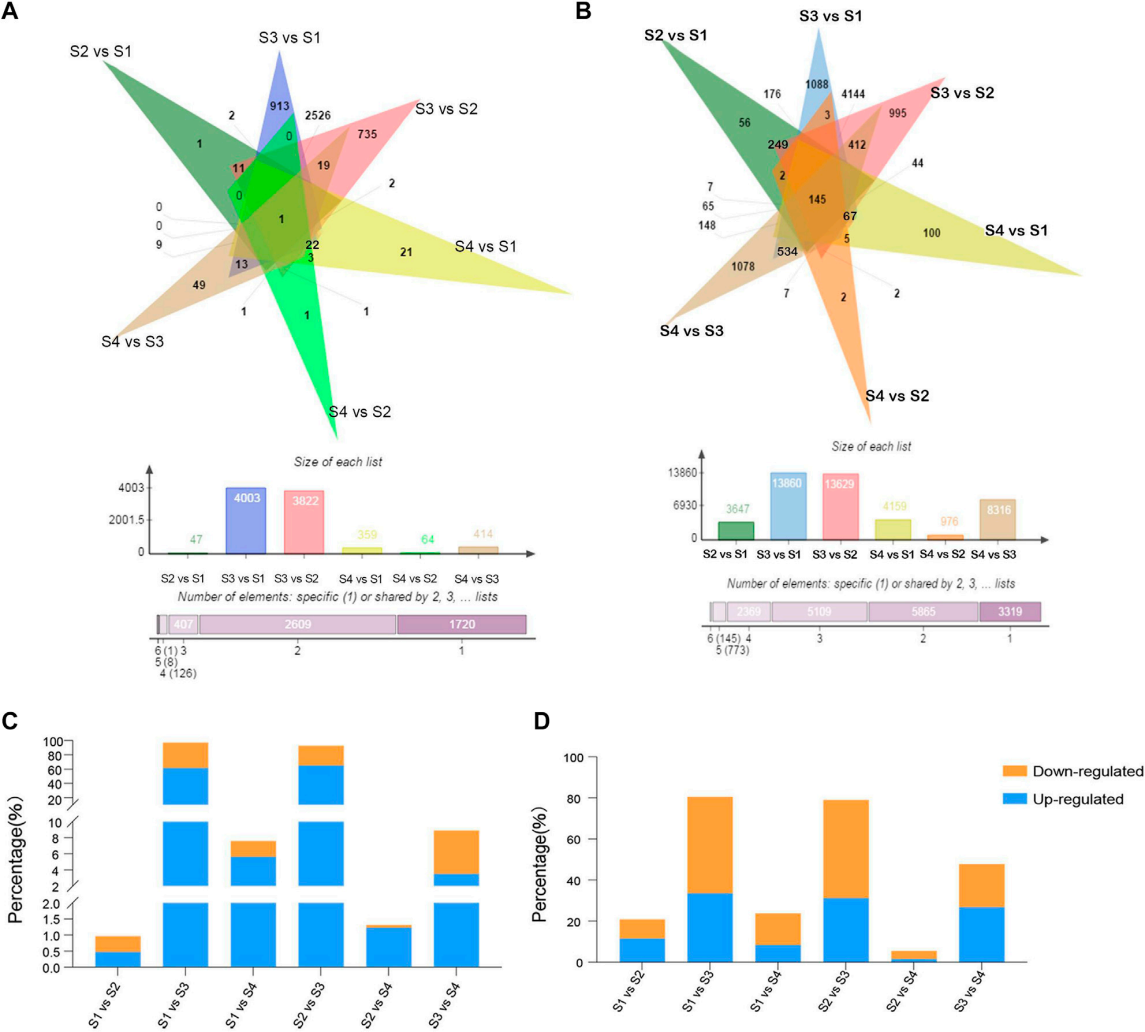
Analysis and identification of differentially expressed lncRNAs (DELs). **(A)** Venn diagram of common and specific DELs. **(B)** Venn diagram of common and specific differentially expressed mRNAs. **(C,D)** Percentages of DEGs and DELs.

To identify lncRNAs in different developmental stages of blood-fleshed peaches, six comparison groups were analyzed: S1 versus S2, S1 versus S3, S1 versus S4, S2 versus S3, S2 versus S4, and S3 versus S4. Differentially expressed lncRNAs (DELs) were identified for each comparison group and the number of upregulated and downregulated DELs were shown (Supplementary Figures S1A–F). In summary, we obtained 4,871 DELs, of which one was common to these groups (Figure 2A), and 17,580 DEGs, of which 145 were common to the six comparison groups (Figure 2B). Moreover, DEGs were more than DELs in six comparisons, and the percentages of DEGs and DELs in S3 versus S1 and S3 versus S2 were higher (Figures 2C,D).

Specifically, 4,515 DELs were differentially expressed in fruits at S3 versus S2, of which 3,168 were upregulated and 1,347 were downregulated. A comparison of the fruits at S4 and S3 revealed significant variations in the expression. A total of 434 DELs, comprising 170 upregulated and 264 downregulated genes, were identified in the fruits at S3 (Supplementary Table S3).

**FIGURE 3:**
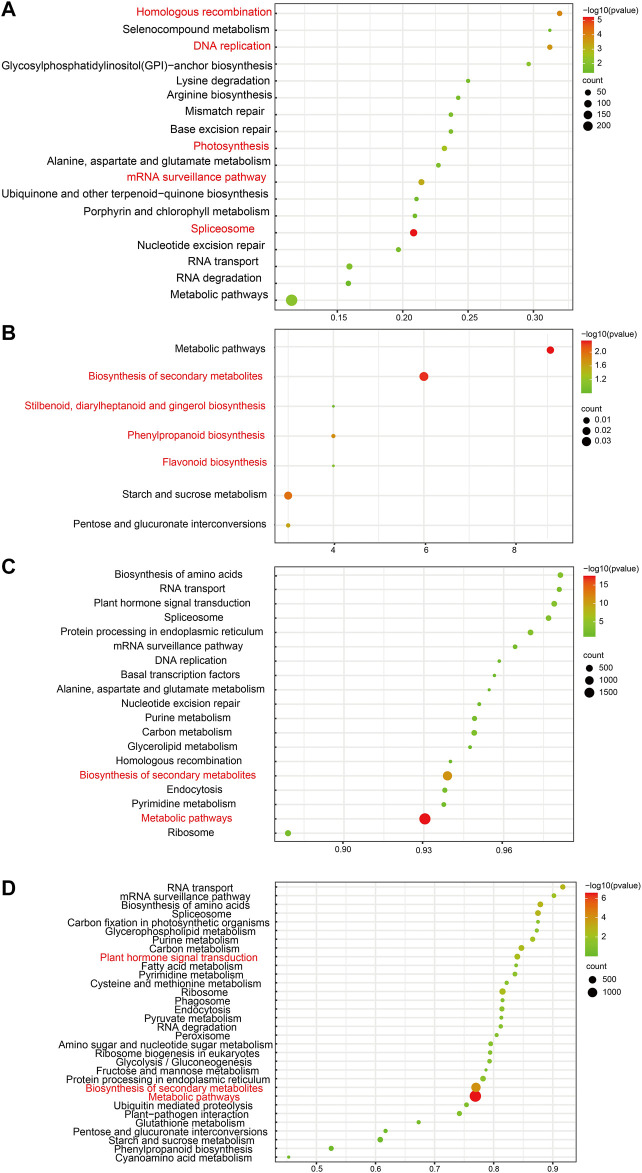
Common and unique enriched KEGG pathways of four developmental stages. **(A)** Common enriched KEGG pathways of four stages with trans-regulation function. **(B)** Common enriched KEGG pathways of four stages with cis-regulation function. **(C)** Unique enriched GO terms of four stages with trans-regulation function. **(D)** Unique enriched GO terms of four stages with cis-regulation function.

The number of shared and exclusive DELs between the different developmental stages of the blood-fleshed peach is shown by the Venn diagram (Figure 3A). Only one of the DELs was expressed in all developmental stages, while one (2.1%) DEL was exclusively expressed between the S2 and S1 stages, 913 (30.5%) between the S3 and S1 stages, 21 (8.7%) between the S4 and S1 stages, 735 (23.2%) between stages S3 and S2, 1 (1.7%) between stages S4 and S2, and 49 (28.9%) between stages S4 and S3. S3 versus S1 stages had the highest number of DELs.

We mapped these lncRNAs and their target transcript mRNAs onto eight chromosomes of the peach genome and found that some lncRNAs were produced at the ends of chromosomes #6 and #3 (Supplementary Figure S2).

### Expression Correlation of DE-lncRNAs With *Cis-*acting Protein-Coding Genes

We predicted the potential function of lncRNAs in peach fruit to determine whether protein-coding genes could interact with lncRNAs in *cis* or *trans* configurations. Several DE-lncRNAs have been shown to regulate the expression of genes in close proximity (*cis*-acting) or at a distance (*trans*-acting). In total, 67,232 lncRNA–mRNA pairs were co-localized, with 25,846 lncRNAs upstream and 27,008 lncRNAs downstream of mRNAs, 14,375 antisense lncRNAs, and three sense overlapping (10 kb) with *cis* lncRNA targets that were mainly represented in the developmental process. The most relevant GO terms that were associated with unique biological processes and molecular functions contained many key enzymes and important response regulators, consistent with the steps involved in fruit development stages (Supplementary Table S5). These analyses identified *cis-*acting lncRNAs with potential regulatory roles.

The main KEGG pathways of *cis*-target genes associated with fruit development include stilbenoid, diarylheptanoid, gingerol, flavonoid, phenylpropanoid, and secondary metabolite biosynthesis, which were enriched with high reliability (Figure 3B).

### Expression Correlation of DE-lncRNAs With *Trans-*acting Protein-Coding Genes

We also found the lncRNAs and mRNAs with *trans*-regulatory relationships from our analyses. A total of 1,048,575 lncRNA–mRNA were co-expressed and 225,537 and 823,038 lncRNAs were positively and negatively correlated with that of their target mRNAs, respectively.

Our functional prediction is based on the GO-term enrichment of *trans* lncRNA targets for biological processes, cellular components, and molecular functions. We compared common and uniquely enriched GO terms in the four stages (Supplementary Table S4). These results suggested that the DE-lncRNAs’ *trans* target genes are involved in a lot of biological processes, such as metabolic processes, cellular processes, and biological regulation, and a variety of molecular functions, such as catalytic activity, binding, transporter activity, and molecular function regulation. These results also suggested that lncRNAs can play important roles in transport and regulation. We identified a series of target genes involved in UV-B-induced anthocyanin biosynthesis in blood-fleshed peaches based on pathway analysis.

Moreover, the KEGG pathway analysis showed that these *trans* target genes of lncRNAs were enriched in spliceosomes, homologous recombination, DNA replication, mRNA surveillance, and photosynthesis pathways (Figure 3A). It indicated that lncRNAs take an essential part in RNA splicing and photosynthesis pathways during fruit development and UV-B-induced anthocyanin biosynthesis in the flesh. This phenomenon illustrated the capability of lncRNAs to affect photosynthesis, which is predominantly involved in fruit development and flesh-coloring processes.

Enriched GO terms of the *cis*- and *trans*-target genes of unique DE-lncRNAs in each of the four development stages differed in the number of involved genes. Still, the enrichment mainly showed some biological processes (developmental, cellular, metabolic, and organic substances) and molecular functions, such as catalytic activity and binding. In addition, the number of genes in *cis*-and *trans*-target unique gene GO terms was roughly similar. Unique DE-lncRNAs’ trans-target genes enrich KEGG pathways at each stage of development, indicating that there is the highest reliability of metabolic pathways and biosynthesis of secondary metabolites (Figure 3C). Unique DE-lncRNAs’ cis-target genes are enriched in KEGG pathways at each developmental stage, suggesting that metabolic pathways, secondary metabolite biosynthesis, and plant hormone signal transduction have the highest reliability (Figure 3D). These results indicated that unique DE-lncRNAs in each developmental stage may play different roles but are involved in the same fruit development processes.

### DE-lncRNAs Participate in lncRNA–mRNA Interactive Networks

**FIGURE 4:**
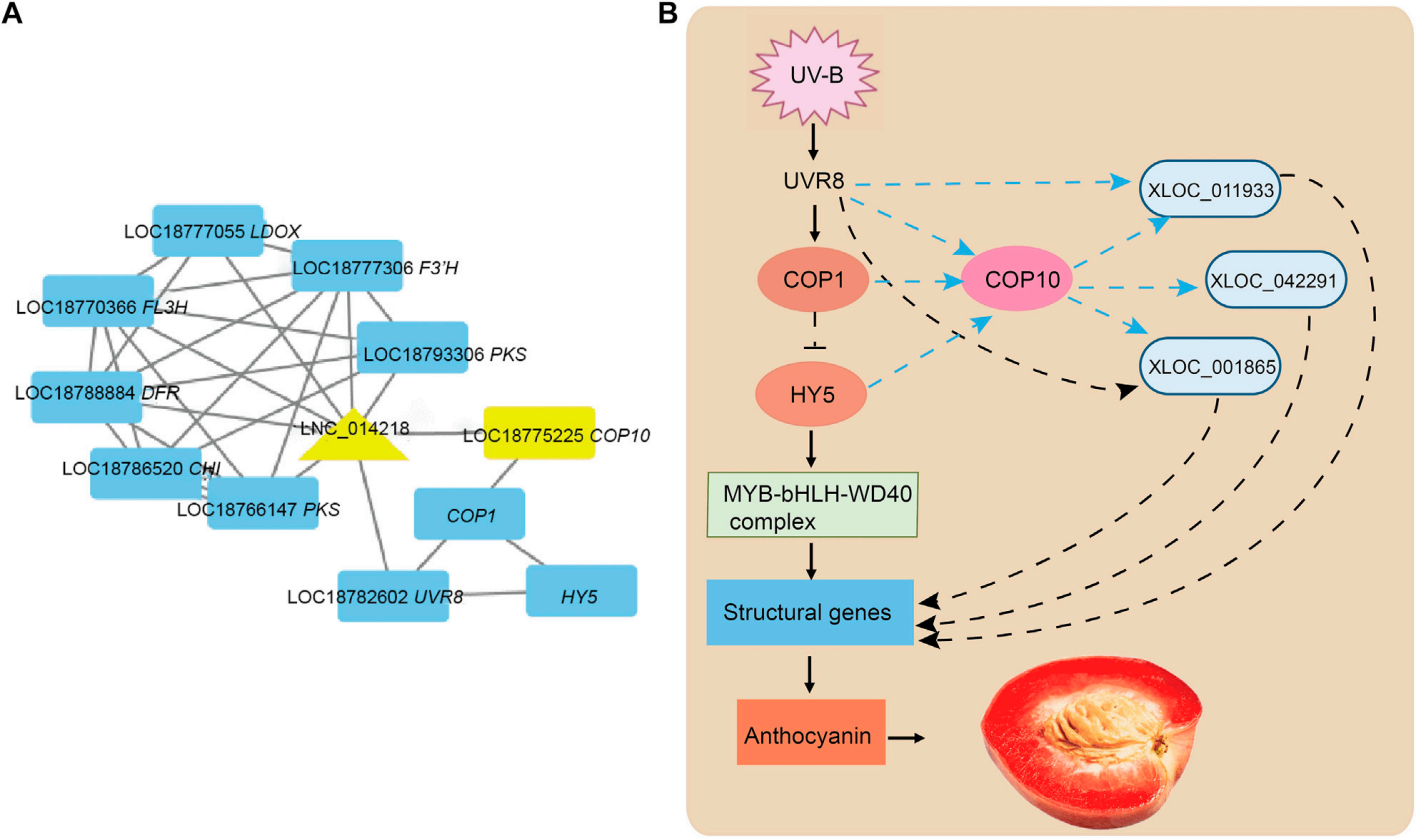
Analysis of XLOC_042291, XLOC_011933, and XLOC_001865 involved lncRNA–mRNA networks associated with UV-B induced in anthocyanin biosynthesis. **(A)** Sub-interaction networks of XLOC_042291, XLOC_011933, and XLOC_001865 from lncRNA–mRNA networks. The triangle represents lncRNAs and the rectangle represents mRNAs. **(B)** Model diagram of UV-light-mediated fruit development of red meat peach. *COP1*, constitutive photomorphogenic 1; *COP10*, constitutive photomorphogenic 10; *HY5*, ELONGATED HYPOCOTYL 5; *UVR8*, UV RESISTANCE LOCUS 8; Ub, ubiquitin.

Next, we elucidated the function of DE-lncRNAs and the relationship between lncRNAs and mRNAs that were co-expressed and fell <10 kb away from DE-lncRNAs by establishing putative interactive networks using Cytoscape. Three anthocyanin biosynthesis-related lncRNAs (XLOC_011933, XLOC_001865, and XLOC_042291) were identified for further analysis (Figure 4A). The transcriptome data of identified lncRNA genes and transcription factors were analyzed. A model diagram on the UV-B regulation of peach anthocyanin synthesis was presented (Figure 4B). *COP1* and *COP10* are involved in the degradation of downstream genes, including members of the *HY5*, *HYH,* and *MBW* complex. The identified lncRNAs interacted with some structural genes as critical regulators playing particular roles in UV-B-induced anthocyanin biosynthesis. The results indicated that the three lncRNAs could affect biological processes at different levels.

### Analysis of lncRNAs Related to the UV-B-Induced Anthocyanin Biosynthesis of Fruits

**FIGURE 5:**
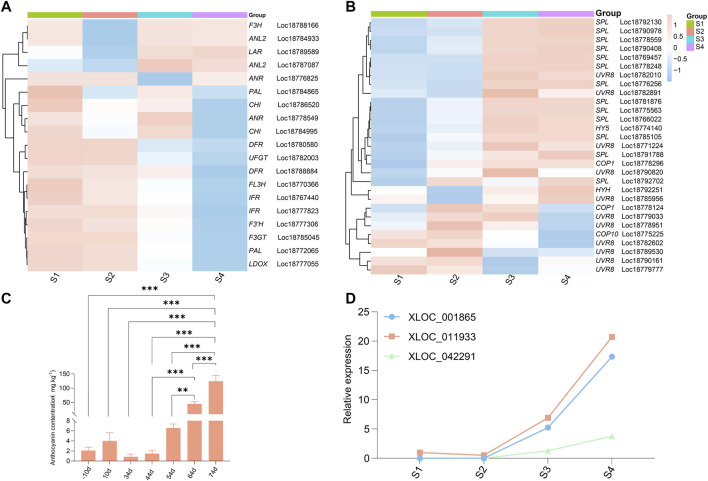
Heatmap and graphical presentations of selected genes during fruit development stages. **(A)** Expression profiles of structural genes involved in the anthocyanin biosynthesis pathway. The color gradient with eight different colors from blue to red corresponds to transcript levels from low to high, with the values representing the log2 (FPKM) values. **(B)** Expression profiles of genes related to UV-light-mediated anthocyanin biosynthesis. The color key at the top of the heatmap represents the relative color scheme of the FPKM values. **(C)** Anthocyanin content in different development stages. **(D)** Graphical representations of XLOC_011933, XLOC_001865, and XLOC_042291 related to anthocyanin biosynthesis based on RNA-seq values in blood-fleshed peach.

We also investigated the cross-talk among lncRNAs, *COP10,* and *UVR8* in UV-B-induced anthocyanin accumulation in the blood-fleshed peach fruit development process. Based on the results of preliminary GO, KEGG, and functional analyses, the expression of the three identified lncRNAs XLOC_011,933, XLOC_001,865, and XLOC_042,291, and some structural genes, such as *PAL, DFR, FL3H, F3GT, LDOX,* and *UFGT,* and plant photoreceptors *UVR8* (UVB photoreceptor), downstream signal elements *COP10, COP1, HY5, HYH* (*HY5* homolog), and *SPL* were analyzed. The results revealed that most structural genes showed similar expression patterns, corroborating the accumulation of anthocyanins, except for *ANL2* (Loc18784933 and Loc18787087), *LAR* (Loc18789589), and *F3H* (Loc18788166) (Figure 5A). For the genes that regulate light signaling, only *COP10* (Loc18775225) and *UVR8* (Loc18782602) showed similar expression patterns with the accumulation of anthocyanins, suggesting a relation between their regulation and respective functions (Figure 5B).

Our analyses revealed 12 light receptors (*UVR8s*, eight LIGHT-DEPENDENT SHORT HYPOCOTYLS, and four constitutive photomorphogenesis proteins (*COP*)), as derived using automated computational analysis in the peach genome. *UVR8s* and *SPLs* were distributed on different chromosomes and displayed different expression patterns during the four fruit development stages (Figures 5A,B). This observation implied that these photoreceptors and signal elements have different functions among molecules, although they belong to the same gene family.

These results suggested lncRNAs’ involvement in regulating anthocyanin biosynthesis and fruit flesh pigment in developing blood-fleshed fruits.

### Examination of the Evolutionary Conservation of lncRNAs

We hypothesized that if lncRNAs are evolutionarily conserved, they could perform similar functions in different species even without coding regions (Kang and Liu, 2015). Naturally, we tested whether the three lncRNAs found in this study are evolutionarily conserved in different plant species. NCBI BLAST analysis of the three lncRNA sequences revealed that XLOC_001865 shares high protein sequence similarities with *Prunus persica* aquaporin *TIP1-2* (LOC18767586), *Prunus mume* aquaporin *TIP1-2-like* (LOC103335008), and *Prunus avium* aquaporin *PIP-type-like* (LOC110757218). Two conserved domains of XLOC_001865 (507 bp), one of which belongs to the major intrinsic protein (*MIP*) superfamily, were conserved with the aforementioned three proteins, implying that this region may have potentially conserved counterparts in the Rosaceae species. Analysis of the gene sequence of XLOC_011933 (4,272 bp) revealed that a 382 bp fragment shared 100% identity with the predicted *P*r*unus avium* chitinase 2-like (LOC110760813) DNA sequence and revealed an 868 bp repeat sequence at the beginning and interval of the lncRNA sequence. The BLAST analysis revealed that XLOC_042291 (1945 bp) shared no similarity with any known proteins or DNA sequences but with some uncharacterized mRNA or ncRNA in the peach genome. This analysis failed to determine whether the homologous sequences in the other species encoded lncRNAs, suggesting limited evolutionary conservation of the lncRNAs.

### Reverse-Transcription Quantitative PCR

**FIGURE 6:**
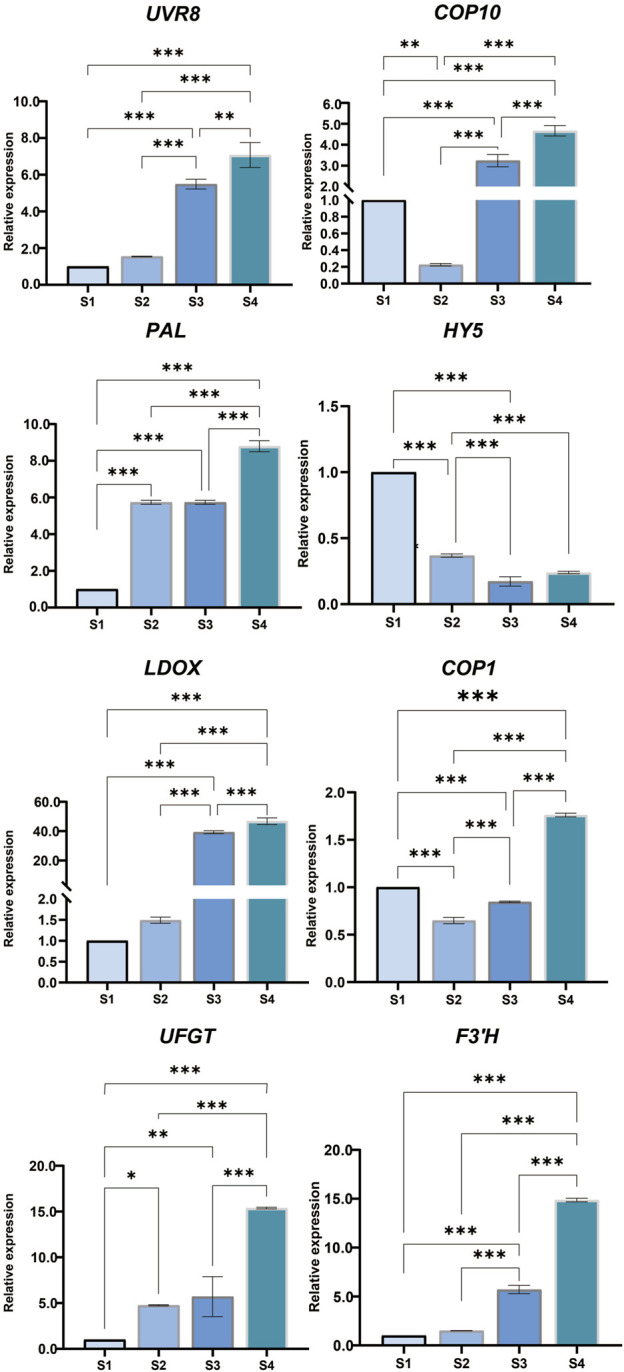
Quantitative real-time (qRT)-PCR was used to determine anthocyanin biosynthesis-related genes *UVR8* (Loc18782602)*, UFGT* (Loc18782003)*, PAL* (Loc18772065)*, LDOX* (Loc18777055)*, HY5* (Loc18774140)*, F3 ′H* (Loc18777306)*, COP10* (Loc18775225)*,* and *COP1* (Loc18778124) in peach fruit. The error bar represents the mean + standard error of three repeated measurements (SE) using one-way analysis of variance (ANOVA). *p* < 0.05 is represented by * and *p* < 0.001 is represented by ***.

The expression trends of some genes, including *COP10* (LOC18775225), *HY5* (LOC18774140), *UVR8* (LOC18782602), *COP1* (LOC18778124), *PAL* (LOC18772065), *F3′H* (LOC18777306), *UFGT* (LOC18782003), and *LDOX* (LOC18777055), were examined. These results were consistent with the trend of FPKM (Figure 6).

## Discussion

Although extensive studies have described the physiological and molecular aspects of peach development and ripening, few studies have focused on lncRNA-based molecular regulation in controlling anthocyanin biosynthesis and flesh coloration during peach fruit development. In this study, we have attempted to address such missing information by exploring peach development and ripening aspects based on lncRNA-associated mechanisms. No lncRNA has been described as playing a role in peach development; therefore, lncRNAs are associated with flesh color. We performed genome-wide investigations based on sequencing to identify lncRNAs playing a role in *Prunus persica*, thereby providing a new perspective for studying the regulation mechanism of non-coding genes regulatory mechanisms in peach genomes.

Through transcriptome data analysis, stringent filtering criteria mining transcriptome datasets of these lncRNAs were applied. A total of 4,871 differentially expressed lncRNAs and 17,580 DEGs were screened. Also, regulating target genes in two ways such as *trans* and *cis* could provide valuable methods for identifying the processes involved in lncRNAs and inferring their potential functions. Increasing evidence points out that lncRNAs play indispensable roles in plant responses and the regulation of secondary metabolism (An et al., 2018; Yin et al., 2018). RNA-seq analysis of the blood-fleshed peach revealed numerous lncRNAs involved in various biological processes. In this examination, potential *cis* and *trans* lncRNA target genes and their functions were predicted and analyzed by us according to their relationship with mRNA expression. They were found to be involved in many processes, including flavonoid and anthocyanin biosynthetic pathways.

Studies have shown that the synthesis process of anthocyanin from phenylalanine is complicated, from phenylalanine decomposition to anthocyanin biosynthesis (Jaakola, 2013). It has been reported in many works (Shi and Xie, 2014). We identified three DE-lncRNAs that were significantly involved in anthocyanin biosynthesis pathways. As shown in Table 2, XLOC_001,865 was predicted to target *FL3H, COP10, UFGT, UVR8, DFR,* and *PAL*. XLOC_011,933 was predicted to target *PAL, FL3H, LDOX, F3GT, UFGT, MYB5, DFR, UVR8*, and *COP10*. XLOC_042,291 was predicted to target *PAL, FL3H, LDOX, F3GT, DFR, UFGT, COP10,* and *UVR8*. These results indicated a possible regulatory relationship between lncRNAs and anthocyanin biosynthesis structural gene photoreceptors and light signal transduction-related genes. The expression pattern analysis showed a similar pattern for most structural genes, which paralleled the accumulation of anthocyanins (Figure 5A). *ANL2* (ANTHOCYANINLESS2), which belongs to the *HD–ZIP* family, has been reported to be possibly involved in the accumulation of anthocyanin in *Arabidopsis* (Kubo et al., 1999), exhibiting an opposite trend with an accumulation of anthocyanins.

**TABLE 2:** XLOC_011933, XLOC_001865, and XLOC_042291 regulate anthocyanin biosynthesis in the fruit development of blood-fleshed peach.

lncRNA_ID	mRNA_ID	Predicted product abbreviation	Predicted product	*p*-value
LNC_000563	XM_007202045.2	Naringenin,2-oxoglutarate 3-dioxygenase	*FL3H*	1.21E-07
LNC_000563	XM_007205913.2	Constitutive photomorphogenesis protein 10	*COP10*	2.46E-09
LNC_000563	XM_007211190.2	MYB4	*MYB4*	1.55E-06
LNC_000563	XM_007216787.2	UDP-glucose flavonoid 3-O-glucosyltransferase 3	*UFGT*	5.48E-08
LNC_000563	XM_007217820.2	Ultraviolet-B receptor UVR8	*UVR8*	1.28E-07
LNC_000563	XM_007222255.2	Bifunctional dihydroflavonol 4-reductase/flavanone 4-reductase	*DFR*	3.05E-08
LNC_000563	XM_007208370.2	Phenylalanine ammonia-lyase 1	*PAL*	1.29E-11
LNC_003950	XM_007202045.2	Naringenin,2-oxoglutarate	*FL3H*	1.57E-07
		3-dioxygenase		
LNC_003950	XM_007205236.2	*MYB5*	*MYB5*	6.47E-07
LNC_003950	XM_007205913.2	Constitutive photomorphogenesis protein 10	*COP10*	1.48E-10
LNC_003950	XM_007208370.2	Phenylalanine ammonia-lyase	*PAL*	5.34E-11
LNC_003950	XM_007210458.2	Leucoanthocyanidin dioxygenase	*LDOX*	8.26E-10
LNC_003950	XM_007215227.2	UDP-glucose flavonoid	*UFGT*	2.21E-06
		3-O-glucosyltransferase 3		
LNC_003950	XM_007217820.2	Ultraviolet-B receptor UVR8	*UVR8*	5.80E-07
LNC_003950	XM_007217890.2	Anthocyanidin 3-O-glucosyltransferase 2	*F3GT*	3.57E-10
LNC_003950	XM_007222255.2	Bifunctional dihydroflavonol 4-reductase/flavanone 4-reductase	*DFR*	3.84E-08
LNC_014218	XM_007202045.2	Naringenin,2-oxoglutarate 3-dioxygenase	*FL3H*	5.85E-08
LNC_014218	XM_007205913.2	Constitutive photomorphogenesis protein 10	*COP10*	2.32E-09
LNC_014218	XM_007208370.2	Phenylalanine ammonia-lyase	*PAL*	1.53E-12
LNC_014218	XM_007210458.2	Leucoanthocyanidin dioxygenase	*LDOX*	1.48E-10
LNC_014218	XM_007215227.2	UDP-glucose flavonoid 3-O-glucosyltransferase 3	*UFGT*	7.08E-07
LNC_014218	XM_007217820.2	Ultraviolet-B receptor UVR8	*UVR8*	1.12E-07
LNC_014218	XM_007217890.2	Anthocyanidin 3-O-glucosyltransferase 2	*F3GT*	1.95E-12
LNC_014218	XM_007222255.2	Bifunctional dihydroflavonol 4-reductase/flavanone 4-reductase	*DFR*	1.34E-07

The italics refer to the target genes predicted by the three DE-LNcRNAs (XLOC_001865, XLOC_011933, and XLOC_042291).

In this research, we also identified the conservation of XLOC_001865, XLOC_011933, and XLOC_042291, and found that the evolutionary conservation of lncRNAs could be limited. This result was consistent with the results of lncRNA-related studies (Liu et al., 2012). In addition, we conducted q-PCR verification on the screened structural genes and photoreceptor-related genes and found that their expression trends were consistent with transcriptome data, proving that the transcriptome data were reliable. The results showed that these differentially expressed long non-coding genes played specific roles in the anthocyanin biosynthesis of peach fruits.

Peach is a rich genetic resource, in which the discovery of lncRNAs will bring great convenience to breeding. Rosaceae is a branch of fruit trees, and it is very important to study its fruit. Thus, understanding the lncRNA-mediated network regulation of UV-B-induced anthocyanin biosynthesis would lay the foundation for unraveling the complex molecular genetic mechanisms of positive effects on improving agronomic traits.

## Conclusion

Color is one of the most essential sensory attributes of fresh fruits, and it influences the choices and preferences of consumers, indicates maturity, and correlates with other quality attributes, such as sugar and acid content and flavor. Water-soluble anthocyanins can produce different colors, such as red, purple, and blue. In the present study, we sought to identify lncRNAs from fruit transcriptomes to identify lncRNAs that function in fruit development. We identified and screened some differentially expressed lncRNAs by the transcriptome analysis. Through fluorescence real-time quantitative PCR (qRT-PCR) experiments, we found that the results were the same as the trend of the transcriptome. This study is the first to analyze and discover the lncRNAs involved in fruit coloration in peaches. The findings from this study may encourage researchers to study peach flesh coloring in detail.

## Data Availability

The datasets presented in this study can be found in online repositories. The names of the repository/repositories and accession number(s) can be found in the article/Supplementary Material.
