# Supplementary material for: Identification and Analysis of Long Non-Coding RNAs Related to UV-B-Induced Anthocyanin Biosynthesis During Blood-Fleshed Peach (Prunus persica) Ripening
Source: Front Genet. 2022 Aug 9;13:932207. doi: 10.3389/fgene.2022.932207 (PMC9395590; doi:10.3389/fgene.2022.932207)
Supplement: Supplementary file 1 [file DataSheet1.PDF]

Table S1 RNA-seq filters data

Sample name	S1_11	S1_12	S1_13	S2_21	S2_22	S2_23	S3_31	S3_32	S3_33	S4_41	S4_42	S4_43
Total reads	100842110	105585708	96262152	103705758	113957100	95926382	102758664	83870212	96244550	106839978	103571376	97653486
Total mapped	82313947 (81.63%)	85118068 (80.62%)	78831939 (81.89%)	87513785 (84.39%)	94876985 (83.26%)	79562723 (82.94%)	89683195 (87.28%)	72940253 (86.97%)	83972920 (87.25%)	96488513 (90.31%)	93928354 (90.69%)	88337685 (90.46%)
Multiple mapped	1537614 (1.52%)	1696963 (1.61%)	1502298 (1.56%)	1680591 (1.62%)	1861581 (1.63%)	1579154 (1.65%)	1801840 (1.75%)	1429730 (1.7%)	1691789 (1.76%)	2315829 (2.17%)	2379863 (2.3%)	2202720 (2.26%)
Uniquely mapped	80776333 (80.1%)	83421105 (79.01%)	77329641 (80.33%)	85833194 (82.77%)	93015404 (81.62%)	77983569 (81.3%)	87881355 (85.52%)	71510523 (85.26%)	82281131 (85.49%)	94172684 (88.14%)	91548491 (88.39%)	86134965 (88.2%)
Read-1	40814313 (40.47%)	42065079 (39.84%)	38956156 (40.47%)	43211030 (41.67%)	46815124 (41.08%)	39205810 (40.87%)	44248842 (43.06%)	35925745 (42.83%)	41346310 (42.96%)	47313519 (44.28%)	46032835 (44.45%)	43318288 (44.36%)
Read-2	39962020 (39.63%)	41356026 (39.17%)	38373485 (39.86%)	42622164 (41.1%)	46200280 (40.54%)	38777759 (40.42%)	43632513 (42.46%)	35584778 (42.43%)	40934821 (42.53%)	46859165 (43.86%)	45515656 (43.95%)	42816677 (43.85%)
Reads map to '+'	40410062 (40.07%)	41610347 (39.41%)	38698031 (40.2%)	42867035 (41.34%)	46455840 (40.77%)	38942052 (40.6%)	43918318 (42.74%)	35734299 (42.61%)	41115717 (42.72%)	47079520 (44.07%)	45767345 (44.19%)	43069756 (44.1%)
Reads map to '-'	40366271 (40.03%)	41810758 (39.6%)	38631610 (40.13%)	42966159 (41.43%)	46559564 (40.86%)	39041517 (40.7%)	43963037 (42.78%)	35776224 (42.66%)	41165414 (42.77%)	47093164 (44.08%)	45781146 (44.2%)	43065209 (44.1%)
Non-splice reads	55869605 (55.4%)	57486737 (54.45%)	53432569 (55.51%)	58036744 (55.96%)	63193142 (55.45%)	53242155 (55.5%)	59503930 (57.91%)	48645543 (58%)	55558797 (57.73%)	61832796 (57.87%)	59797444 (57.74%)	56266702 (57.62%)
Splice reads	24906728 (24.7%)	25934368 (24.56%)	23897072 (24.82%)	27796450 (26.8%)	29822262 (26.17%)	24741414 (25.79%)	28377425 (27.62%)	22864980 (27.26%)	26722334 (27.77%)	32339888 (30.27%)	31751047 (30.66%)	29868263 (30.59%)
Reads mapped in proper pairs	77474772 (76.83%)	79583726 (75.37%)	74186902 (77.07%)	82516616 (79.57%)	89291236 (78.36%)	74909370 (78.09%)	84987774 (82.71%)	69344284 (82.68%)	79815728 (82.93%)	91875122 (85.99%)	89183242 (86.11%)	83835140 (85.85%)

Table S2 Classification of Mapped Reads

Sample	protein_coding	others
S1_11	78.1%	21.90%
S1_12	77.7%	22.30%
S1_13	78.0%	22.00%
S2_21	81.1%	18.90%
S2_22	80.2%	19.80%
S2_23	78.8%	21.20%
S3_31	84.2%	15.80%
S3_32	84.6%	15.40%
S3_33	83.4%	16.60%
S4_41	87.6%	12.40%
S4_42	86.2%	13.80%

Table S3 Differentially Expressed lncRNAs

LncRNA	Up-regulated	Down-regulated	Total	Up-regulated	Down-regulated
S1 vsS2	23	24	47	0.472182303	0.492711969
S1 vsS3	2990	1749	4739	61.38369945	35.90638473
S1 vs S4	275	96	371	5.645657976	1.970847875
S2 vs S3	3168	1347	4515	65.03797988	27.65345925
S2 vs S4	60	4	64	1.231779922	0.082118661
S3 vs S4	170	264	434	3.490043112	5.419831657

Table S4 Common enriched GO terms of four stages with trans-regulation function.

	GO term	subgroup	percentage of genes （%）	number of genes
GO:0008152	metabolic process	Biological Process	40.2	1070
GO:0009987	cellular process	Biological Process	42.9	1144
GO:0051179	localization	Biological Process	11	293
GO:0050789	regulation of biological process	Biological Process	13.5	360
GO:0065007	biological regulation	Biological Process	14.3	381
GO:0032502	developmental process	Biological Process	1.9	51
GO:0032501	multicellular organismal process	Biological Process	2.5	67
GO:0000003	reproduction	Biological Process	1.5	39
GO:0051704	multi-organism process	Biological Process	4.2	112
GO:0022414	reproductive process	Biological Process	1.2	33
GO:0050896	response to stimulus	Biological Process	9.1	242
GO:0048519	negative regulation of biological process	Biological Process	1	27
GO:0002376	immune system process	Biological Process	0.7	19
GO:0048518	positive regulation of biological process	Biological Process	1	27
GO:0023052	signaling	Biological Process	4.1	110
GO:0071840	cellular component organization or biogenesis	Biological Process	7.2	191
GO:0040011	locomotion	Biological Process	0.8	21
GO:0098754	detoxification	Biological Process	0	1
GO:0019740	nitrogen utilization	Biological Process	0	1
GO:0040007	growth	Biological Process	0.1	2
GO:0016265	obsolete death	Biological Process	0.2	6
GO:0022610	biological adhesion	Biological Process	0.8	20
GO:0048610	obsolete cellular process involved in reproduction	Biological Process	0.8	22
GO:0070271	obsolete protein complex biogenesis	Biological Process	1.6	43
GO:0007610	behavior	Biological Process	0	1
GO:0072668	obsolete tubulin complex biogenesis	Biological Process	0.1	2
GO:0097034	obsolete mitochondrial respiratory chain complex IV biogenesis	Biological Process	0.1	2
GO:0044464	cell part	Cellular Component	20	534
GO:0005623	cell	Cellular Component	20	534
GO:0043226	organelle	Cellular Component	14.5	386
GO:0044422	organelle part	Cellular Component	7.6	203
GO:0032991	protein-containing complex	Cellular Component	10.4	277
GO:0016020	membrane	Cellular Component	16.9	451
GO:0044425	membrane part	Cellular Component	10.1	270
GO:0099080	supramolecular complex	Cellular Component	0.4	10
GO:0030054	cell junction	Cellular Component	0.1	3
GO:0031974	membrane-enclosed lumen	Cellular Component	2.1	56
GO:0044215	other organism	Cellular Component	0.5	13
GO:0044217	other organism part	Cellular Component	0.5	13

GO:0005576	extracellular region	Cellular Component	3.6	96
GO:0044421	extracellular region part	Cellular Component	1.4	37
GO:0070188	obsolete Stn1-Ten1 complex	Cellular Component	0.1	2
GO:0019012	virion	Cellular Component	2.2	59
GO:0044423	virion part	Cellular Component	2	54
GO:0045202	synapse	Cellular Component	0.1	2
GO:0003824	catalytic activity	Molecular Function	39.3	1048
GO:0005488	binding	Molecular Function	49.9	1329
GO:0005215	transporter activity	Molecular Function	4.7	125
GO:0098772	molecular function regulator	Molecular Function	2.8	74
GO:0060089	molecular transducer activity	Molecular Function	1.1	29
GO:0016209	antioxidant activity	Molecular Function	0.3	7
GO:0045182	translation regulator activity	Molecular Function	0.5	13
GO:0038024	cargo receptor activity	Molecular Function	0	1
GO:0005198	structural molecule activity	Molecular Function	2.9	78
GO:0045735	nutrient reservoir activity	Molecular Function	0.2	4
GO:0140110	transcription regulator activity	Molecular Function	3	79
GO:0004871	obsolete signal transducer activity	Molecular Function	1.1	29
GO:0001076	obsolete transcription factor activity, RNA polymerase II transcription factor binding	Molecular Function	0.4	11
GO:0000989	obsolete transcription factor activity, transcription factor binding	Molecular Function	0.5	14
GO:0000988	obsolete transcription factor activity, protein binding	Molecular Function	0.8	21
GO:0000990	obsolete transcription factor activity, core RNA polymerase binding	Molecular Function	0.3	7
GO:0003840	obsolete gamma-glutamyltransferase activity	Molecular Function	0	1

Table S5 Common enriched GO terms of four stages with cis-regulation function.

	GO term	subgroup	percentage of genes（%）	number of genes
GO:0051179	localization	Biological Process	11.9	7
GO:0065007	biological regulation	Biological Process	6.8	4
GO:0009987	cellular process	Biological Process	28.8	17
GO:0071840	cellular component organization or biogenesis	Biological Process	3.4	2
GO:0050789	regulation of biological process	Biological Process	6.8	4
GO:0008152	metabolic process	Biological Process	42.4	25
GO:0023052	signaling	Biological Process	1.7	1
GO:0050896	response to stimulus	Biological Process	5.1	3
GO:0048518	positive regulation of biological process	Biological Process	3.4	2
GO:0051704	multi-organism process	Biological Process	1.7	1
GO:0000003	reproduction	Biological Process	1.7	1
GO:0022414	reproductive process	Biological Process	1.7	1
GO:0005623	cell	Cellular Component	6.8	4
GO:0044464	cell part	Cellular Component	6.8	4
GO:0043226	organelle	Cellular Component	5.1	3
GO:0044422	organelle part	Cellular Component	3.4	2
GO:0031974	membrane-enclosed lumen	Cellular Component	1.7	1
GO:0016020	membrane	Cellular Component	11.9	7
GO:0044425	membrane part	Cellular Component	3.4	2
GO:0032991	protein-containing complex	Cellular Component	3.4	2
GO:0099080	supramolecular complex	Cellular Component	1.7	1
GO:0044423	virion part	Cellular Component	1.7	1
GO:0019012	virion	Cellular Component	1.7	1
GO:0003824	catalytic activity	Molecular Function	44.1	26
GO:0060089	molecular transducer activity	Molecular Function	1.7	1
GO:0005215	transporter activity	Molecular Function	8.5	5
GO:0005198	structural molecule activity	Molecular Function	1.7	1
GO:0005488	binding	Molecular Function	30.5	18
GO:0140110	transcription regulator activity	Molecular Function	3.4	2
GO:0004871	obsolete signal transducer activity	Molecular Function	1.7	1

Table S6. Primer pairs for QRT-PCR

Gene name	primer
COP10-F	TGAGTGTGCTTGACGATGACGAAG
COP10-R	TGAGGACGACACAGAGGTGGTG
UVR8-F	TTGTGTTTGGTTGATGTGCGTATGC
UVR8-R	TCTTCGCTCCTCAGTTCCGTCTC
HY5-F	AGGAGGAGGTGATGGCGATGTC
HY5-R	AAGAGGGGTGAAGGGTGAAAATTGG
COP1-F	GCCCAGTTCCTCAGCACCAATC
COP1-R	ATCACAGTAAGCCGCAGTTCCAAG
PAL-F	TGTTTGGGAGCACGAAAGAGTCAG
PAL-R	AGTAGCCCTGGAGGAGTGTGTTG
F3'H-F	GAGATGATGGTGTTGGCAGGAGTG
F3'H-R	ACAATGGCGGTCAAGAAGGCATC
UFGT-F	CCGAAGGTTGTGAAGGCAGAAGAG
UFGT-R	GACCCACCATCCATCAAGGCTTTC
LDOX-F	AGGGAGGCTGGAGAAGGAAGTTG
LDOX-R	TGTTGTGGAGTATGAAGGTGAGTGC
RPL13-F	GCAGCGACTGAAGACATACAAG
RPL13-R	GGTGGCATTAGCAAGTTCCTC

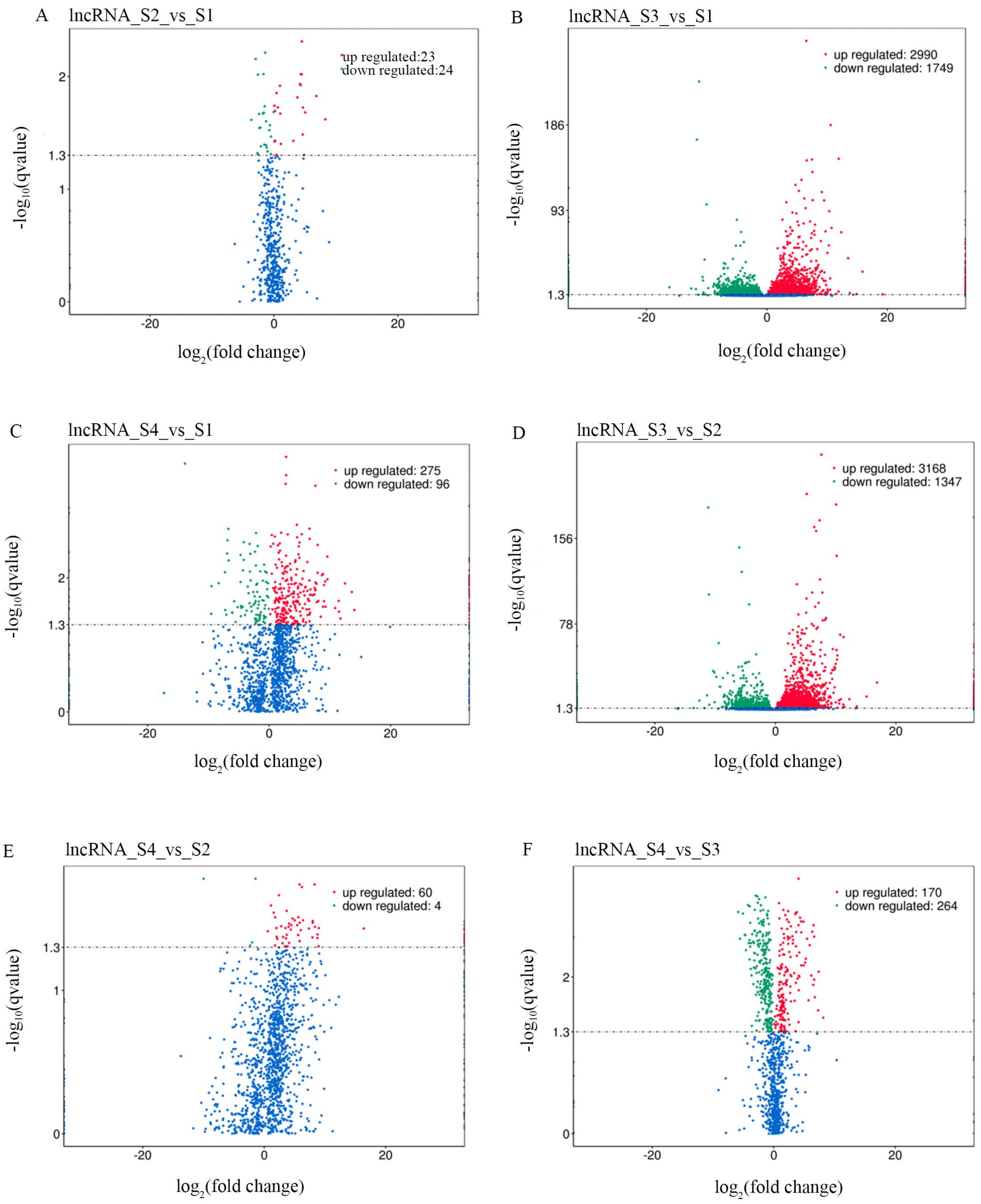

Figure S1 (a–f) Differentially expressed lncRNAs in six comparison groups. Red dots represent significantly up-regulated genes and green dots represent significantly down-regulated genes.

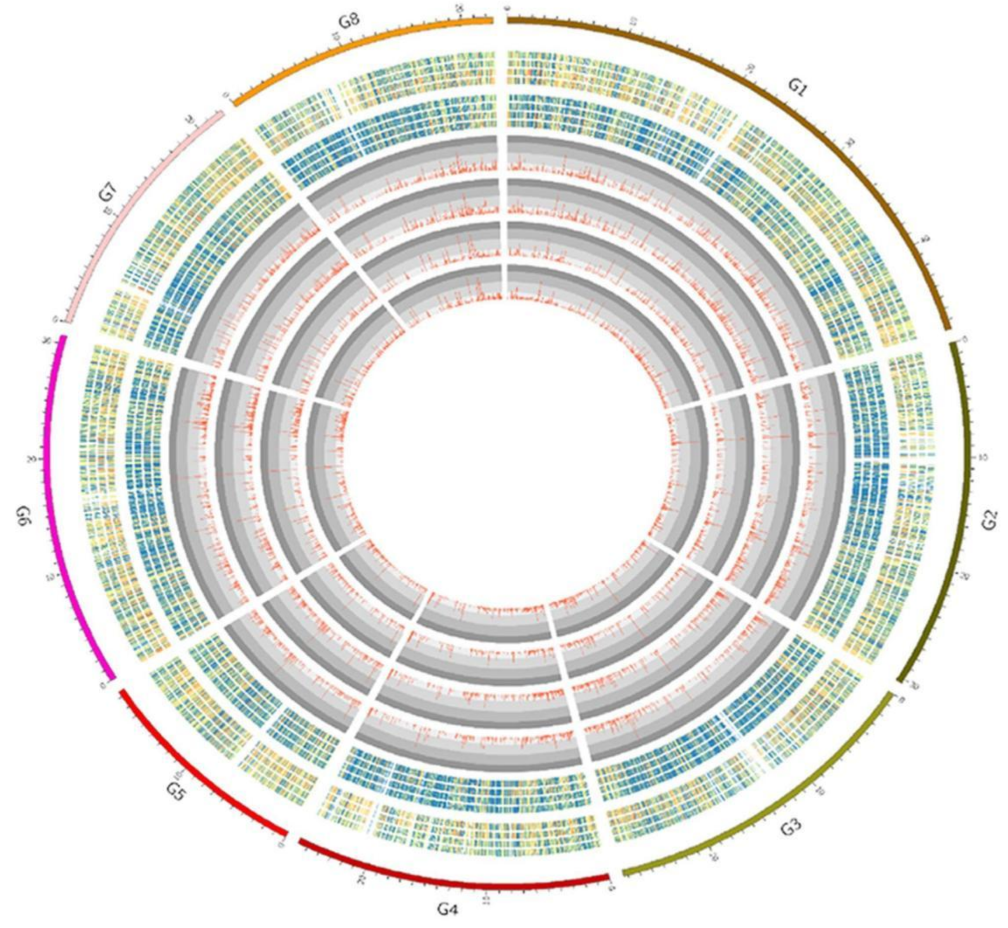

Figure S2 Genome-wide distribution of peach 584 lncRNAs and target transcript mRNA.
